# Unexpected presentation of accessory breast: vulvar accessory breast tissue: a case report

**DOI:** 10.1186/s13256-023-03930-0

**Published:** 2023-05-08

**Authors:** Ghazal Mansouri, Ibrahim Alkatout, Maryam Iranpour, Elham Pourkhandani, Leila Allahqoli

**Affiliations:** 1 Department of Obstetrics and Gynecology, Kerman University of Medical Sciences, Kerman, Iran; 2 University Hospitals Schleswig-Holstein, Campus Kiel, Kiel School of Gynaecological Endoscopy, Arnold-Heller-Str. 3, Haus 24, 24105 Kiel, Germany; 3 Department of Pathology, Pathology and Stem Cell Research Center, Afzalipour Faculty of Medicine, Kerman University of Medical Sciences, Kerman, Iran; 4 Midwifery Department, Ministry of Health and Medical Education, Tehran, Iran; 5 Obstetrics and Gynecology, Kerman University of Medical Sciences, Kerman, Iran

**Keywords:** Accessory breast tissue, Labial region, Case report

## Abstract

**Background:**

The accessory breast is composed of residual glandular mammary tissue that persists after normal embryonic development. The entity is so rare that it is easily neglected in the diagnosis of disease.

**Case presentation:**

We report a 24-year-old virgin Persian woman with a left-sided vulvar mass and no pain or discomfort until shortly before her presentation at our department. Ectopic breast tissue in the vulva was diagnosed. We performed wide local resection of the lesion. Pathological investigation of the lesion confirmed the presence of ectopic breast tissue with secretory changes. She had no specific developmental abnormalities and had no relevant family history. She was followed up for 10 months and had recovered fully by this time.

**Conclusion:**

Accessory breast tissue should be considered as a diagnosis when a mass is seen along the embryonic milk line, especially if the clinical findings reveal changes in the mass accompanied by changes in sex hormones.

## Background

Mammary ridges or milk lines may be described as a bilateral thickening of the ectoderm from the anterior axillary folds to the inguinal folds, which develop into breast tissue during embryogenesis. The only pair that is protected from regression and continues to grow into normal breasts is located in the pectoral region. If the regression process is not completed, accessory breasts may appear [1]. Similar to normal breasts, these ectopic breasts are capable of undergoing physiological and pathological alteration. Unsurprisingly, they also bear the potential of developing cancer. Accessory breast tissue is more common in women than in men, and is known to occur in 0.2–6% of the global population [2]. Accessory breast cancer has been reported in a small number of cases, and the majority of these were observed in the axilla [3]. We describe a rare case of supplementary breast tissue in the vulva.

## Case presentation

**Fig. 1:**
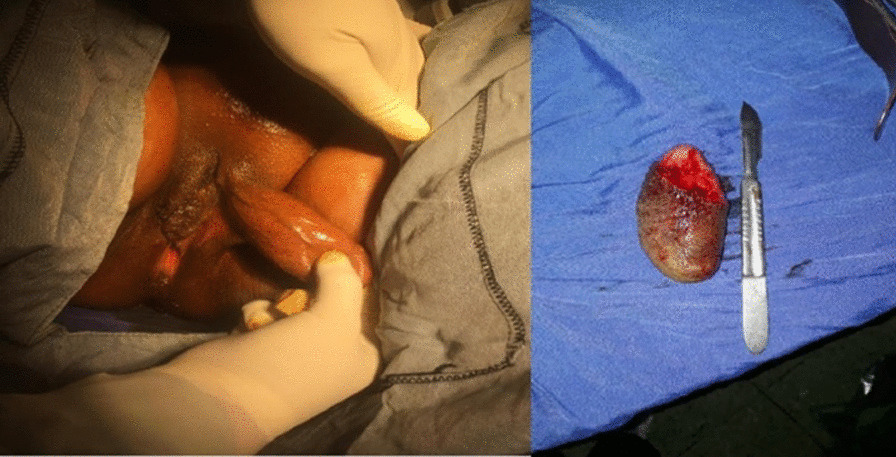
Pedunculated mass in the left labium major

A 24-year-old virgin Persian woman presented with a labial mass that had emerged 12 years earlier and grown gradually in size, but caused no pain or discomfort until recently. Her medical history revealed no evidence of any underlying disease or mental disorder. She had a Bachelor’s degree and was unemployed. Menarche occurred at the age of 11 years, and the patient’s menstrual cycle was regular. She had no family history of breast or gynecological cancer. A pedunculated mass measuring 75 × 44 mm was observed in the upper left labium and looked exactly like a penis. Its texture was relatively soft, the skin temperature normal, and the lesion had clear boundaries with no ulceration (Fig. 1). There was no connection between progressive growth of the lesion and the patient’s menstrual cycle. The lesion was asymptomatic during menstruation. The patient sought no medical advice until she experienced discomfort in the genital area and had difficulty standing up and sitting down. At admission, her vital signs were blood pressure 115/75 mmHg, pulse rate 73 beats per minute, temperature 36.8 °C, and O2 at 99%. Chest examination was normal and she was well oriented. Breast and abdominal examinations were normal. Axillary and supraclavicular lymph nodes could not be palpated. The patient reported no pain on palpation and had no enlarged lymph nodes in the groin.

Ultrasonography of the lesion revealed a hyperechoic mass with clearly defined margins, internal echoes, and fibroglandular tissue measuring 70 × 40 mm in size. On pelvic ultrasound, there was no abnormality in the uterus and the ovaries. Blood, urine, and serology tests were normal.

**Fig. 2:**
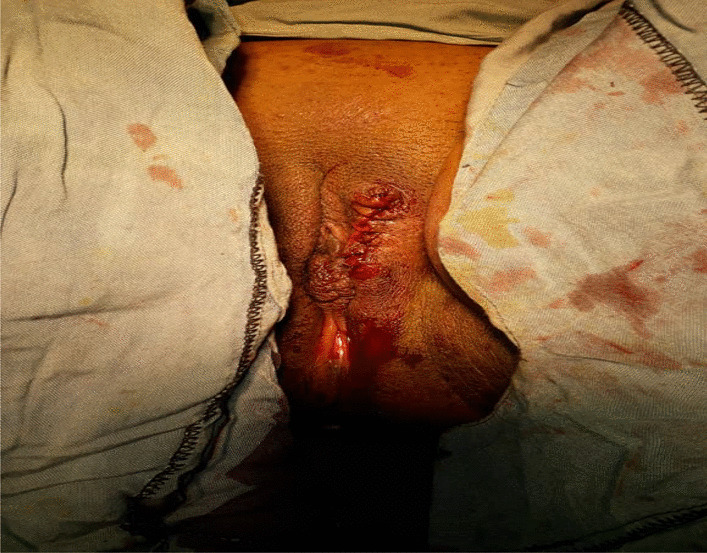
Left labium major after surgery

We decided to perform a resection of the mass, which was then completely removed under local anesthesia and sedation (Fig. 2). The tissue was sent for pathological examination. No postoperative complications were encountered. The patient was discharged from the hospital after a few days.

**Fig. 3:**
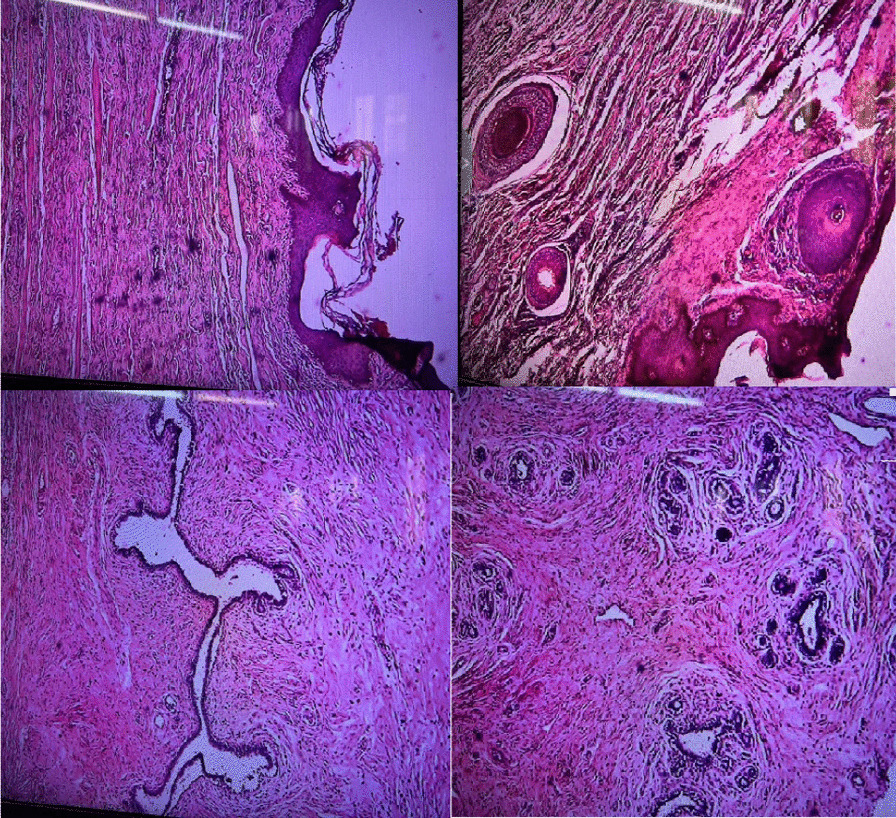
Microphotograph showing breast acini and ducts lined with epithelial and myoepithelial cells [hematoxylin and eosin (H&E) staining, 100-fold magnification]

The pathological investigation revealed a benign lesion. It was an accessory breast composed of female breast tissue, with a proliferative and dilated ductus lined with two layers of endothelial and myoepithelial cells in fibrotic stroma, and invaded by mononuclear inflammatory cells (Fig. 3). She was reviewed in the gynecology outpatient clinic at 2 weeks, 6 weeks, and 10 months post-surgery. Examinations confirmed full recovery with no evidence of recurrence.

## Discussion

We report the case of a 24-year-old virgin woman with vulvar mammary-like tissue resembling a penis and whose origin was unknown. There was no clear hormonal trigger or tumor implicated in the growth. In contrast to the majority of cases described in the published literature, the lesion grew progressively and was in no way connected with neoplastic alterations or oral contraceptive therapy.

Although an accessory breast may be seen at various locations in the body, 60–70% of these occur in the axilla. Accessory breasts have been reported outside the milk line. Some cases have been observed in the perineum, vulva, back, shoulders, and other body parts [4]. The origin of vulvar accessory breast tissue is controversially discussed.

Anogenital mammary-like glands possess characteristics of both eccrine and apocrine glands. It has been suggested that these mammary-like glands, which are highly concentrated in the vulva, resemble vulvar external breast tissue (EBT) rather than milk line remnants [5–7]. EBT of the vulva is occasionally confused with benign subcutaneous processes such as abscess, lipoma, or an epidermoid cyst. The same physiological and pathological changes that affect benign and malignant normal breast tissue may also influence EBT [8]. The latter may operate as a secretory organ because of hormonal changes caused by puberty, pregnancy, or lactation. A typical vulvar EBT manifestation is postpartum tenderness and swelling of the vulva [9–13]. There have been reports of fibroadenoma, fibrocystic alteration, and phyllodes tumor of the vulva [6, 8, 14, 15].

Primary breast carcinoma of the vulva is exceedingly rare in contrast to primary breast cancer, but is treated similarly with excision, lymph node dissection, radiation, chemotherapy, and hormone therapy [16]. Very few occurrences of vulvar or suprapubic involvement have been reported to date (Table 1).

**Table 1:** Review of vulvar accessory breasts without an associated tumor

Author/year	Clinical Manifestation	Age (year)/history	Site	Procedure
Levin and Diener/1968 [17]	Painless enlargement during pregnancy	29/Multiparous	Bilaterally on the labia majora	Partial vulvectomy
Reeves and Kaufman/1980 [18]	Painless enlargement during lactation	29/Multiparous	Left periclitoral location	Surgery
Cobellis *et al*./1997 [19]	Vulvodynia since the age of 10 years	53/Postmenopausal	Bilaterally on the labia majora and in subclitoral location	Surgery
Kapila *et al*./1998 [20]	Enlargement during pregnancy	30/Multiparous	Bilaterally on the labia majora	Watchful waiting
Basu *et al*./2003 [21]	Enlargement during pregnancy and lacteal secretion during postpartum	20/Primigravida	Left perineal location	Surgery
Bardsley and Petterson/2004 [22]	Painless enlargement during pregnancy and disappearance post lactation	31/Multiparous	Right labium major	Surgery
Duvvur *et al*./2007 [23]	Enlargement in the premenstrual phase and decreased in size during menstruation	41/Not reported	Right-sided periclitoral location	Surgery
Pathak and Preston/2007 [24]	Enlargement during pregnancy	31/Multiparous	Abdominal, bilateral axillary, and right-sided vulvar location	Surgery
England/2007 [25]	Size gradually increased to 2 cm	45/Nulliparous	Left labium minor	Surgery
Hong *et al*./2009 [26]	Gradual painful growth in size	18/Nulliparous	Left pubis	Surgery
Mak *et al*./2009 [27]	Periodic growth synchronized with the menstrual cycle, lacteal secretion, and extra nipple	17/Nulliparous	Right pubis	Surgery
Godoy-Gijón *et al*./2012 [11]	Subcutaneous swelling in the left inferior pubic region	24/Nulliparous	Left inferior pubic region	Surgery

The presence of an accessory breast in the vulva is extremely rare. In the present case, the histopathological analysis revealed supplementary breast tissue without any accompanying malignancy. The patient had never been pregnant and had never breastfed an infant. We recommend the inclusion of this entity in the differential diagnosis of vulvar lesions. The lesion itself should be excised due to the risk of malignant conversion of ectopic tissue.

## Conclusion

The present case proved that a mass growing along the milk line should cause the clinician to consider accessory breast tissue.

## Data Availability

All data generated or analyzed during this study are included in the article.

## Abbreviations

mm: Millimeter
EBT: External breast tissue
H&E: Hematoxylin and eosin
